# Towards an Integrative Understanding of Diet–Host–Gut Microbiome Interactions

**DOI:** 10.3389/fimmu.2017.00538

**Published:** 2017-05-08

**Authors:** Mark N. Read, Andrew J. Holmes

**Affiliations:** ^1^The School of Environmental and Life Sciences, The Charles Perkins Centre, The University of Sydney, Sydney, NSW, Australia

**Keywords:** gut microbiome, digestion, diet, metabolite, modeling, host feedback

## Abstract

Over the last 20 years, a sizeable body of research has linked the microbiome and host diet to a remarkable diversity of diseases. Yet, unifying principles of microbiome assembly or function, at levels required to rationally manipulate a specific individual’s microbiome to their benefit, have not emerged. A key driver of both community composition and activity is the host diet, but diet–microbiome interactions cannot be characterized without consideration of host–diet interactions such as appetite and digestion. This becomes even more complex if health outcomes are to be explored, as microbes engage in multiple interactions and feedback pathways with the host. Here, we review these interactions and set forth the need to build conceptual models of the diet–microbiome–host axes that draw out the key principles governing this system’s dynamics. We highlight how “units of response,” characterizations of similarly behaving microbes, do not correlate consistently with microbial sequence relatedness, raising a challenge for relating high-throughput data sets to conceptual models. Furthermore, they are question-specific; responses to resource environment may be captured at higher taxonomic levels, but capturing microbial products that depend on networks of different interacting populations, such as short-chain fatty acid production through anaerobic fermentation, can require consideration of the entire community. We posit that integrative approaches to teasing apart diet–microbe–host interactions will help bridge between experimental data sets and conceptual models and will be of value in formulating predictive models.

## Introduction

Both diet and the gut microbiome are strongly implicated in the global epidemics of chronic diseases, including obesity and diabetes, cardiovascular disease, cancer, autoimmunity, allergies, and asthma (1–5). However, food-intake and microbial response vary over far shorter timescales than chronic disease development. Therefore, to rationally manipulate health through the diet–microbiome axis, we must understand the mechanisms underpinning long-term microbiome outcomes, and how this impacts immune and metabolic functions (6, 7).

Gut microbes influence the host through three broad categories of molecule: cellular structural components that elicit responses via signaling pathways [e.g., microbe-associated molecular patterns (MAMPs)]; metabolites, constituting either signals or nutritional resources to the host; and effectors that are biochemically active (e.g., toxins or enzymes) (8). MAMP types and quantities can be estimated from which species are present and the total population size (live, dormant, and dead cells). However, metabolite and effector production by active cells reflects environmental conditions such as substrate presence. Thus, understanding microbiome impact on host health requires integration of microbial species traits and cell activity with community structure and diet context. Here, we use the term *community state* to refer collectively to those aspects of species composition and metabolic activity that persist over time.

Long-term diet patterns can drive persistent differences in microbial community composition, despite daily variation in food composition and intake rate. For example, in studies where food composition is constant and intake varies (9) and where food composition is systematically varied between groups (9–12), significant between-group microbiome associations are typically seen. Even animals maintained with constant food composition and net intake, but with different fasting and feeding cycles, can develop characteristic differences in their microbiome (13). However, such diet responses are not consistent between studies (14). Mechanistic explanation of this between-study variability is more complex and requires integration of factors beyond diet.

The most obvious mechanisms of food impact on the microbiome are short-term processes such as growth. Ingestion of a new food component or food removal (fasting) can both alter the community state within 24 h (12, 15). However these responses are transient; it is long-term processes that primarily contribute to chronic community state development. For example, in mice fed a high-fat diet, it typically takes >6 weeks for a community state characteristic of diet-induced obesity to appear (16, 17). Here the appearance of “inflammophilic pathobionts” follows development of an inflammatory state and increased intestinal permeability (17). The essential role of host processes was demonstrated through intra-peritoneal anti-inflammatories that reversed chronic microbial changes, despite mice continuing the high-fat diet (17).

Characteristic inter-individual microbiome differences reflect resilience in community structure (tendency to return to a “baseline” state), and long-term dietary shifts can trigger tipping points in this baseline. The emerging concept of *multistability* captures how factors such as inter-microbial interactions, host interactions, diet, microbe immigration, and anti-microbials can together drive a community to different stable states (7, 18, 19). Novel applications of machine learning have revealed that microbiome data contain signals enabling the prediction of human health outcomes (20, 21). However, understanding the origins of this signal and rationally manipulating a beneficial community state requires consideration of multiple dimensions. Nutritional geometry can map biological parameters and outcomes spanning multiple scales over nutritional intake space, by representing dietary dimensions (e.g., macronutrients) as orthogonal axes and plotting individuals based on their dietary intake (22–24). However, mechanistic understanding requires a conceptual framework encompassing key gut ecosystem dimensions.

## Nutrient Dimensions that Influence Microbial Community State

For symbiosis involving a microbial community within an animal nutrition is a highly multifactorial concept. A fundamental driver of each species’ ecological niche within a community is the relative availability of its nutrient requirements over time. But each community member responds differently to how nutrients enter the ecosystem owing to their different requirements. Autotrophs (e.g., plants and many bacteria) have “complete” biosynthetic capability, and their nutrient requirements are expressible as inorganic compounds. Given their more limited biosynthetic ability, animal nutrient requirements must be expressed in terms of foods containing pre-formed organic compounds. Consequently, human nutrition is typically described in terms of sources and quantities of macromolecules that are major sources of energy, essential amino acids, and essential fats. However, the diversity of microbial metabolic strategies requires “microbiome nutrition” to be described more extensively at the chemical level.

**Figure 1:**
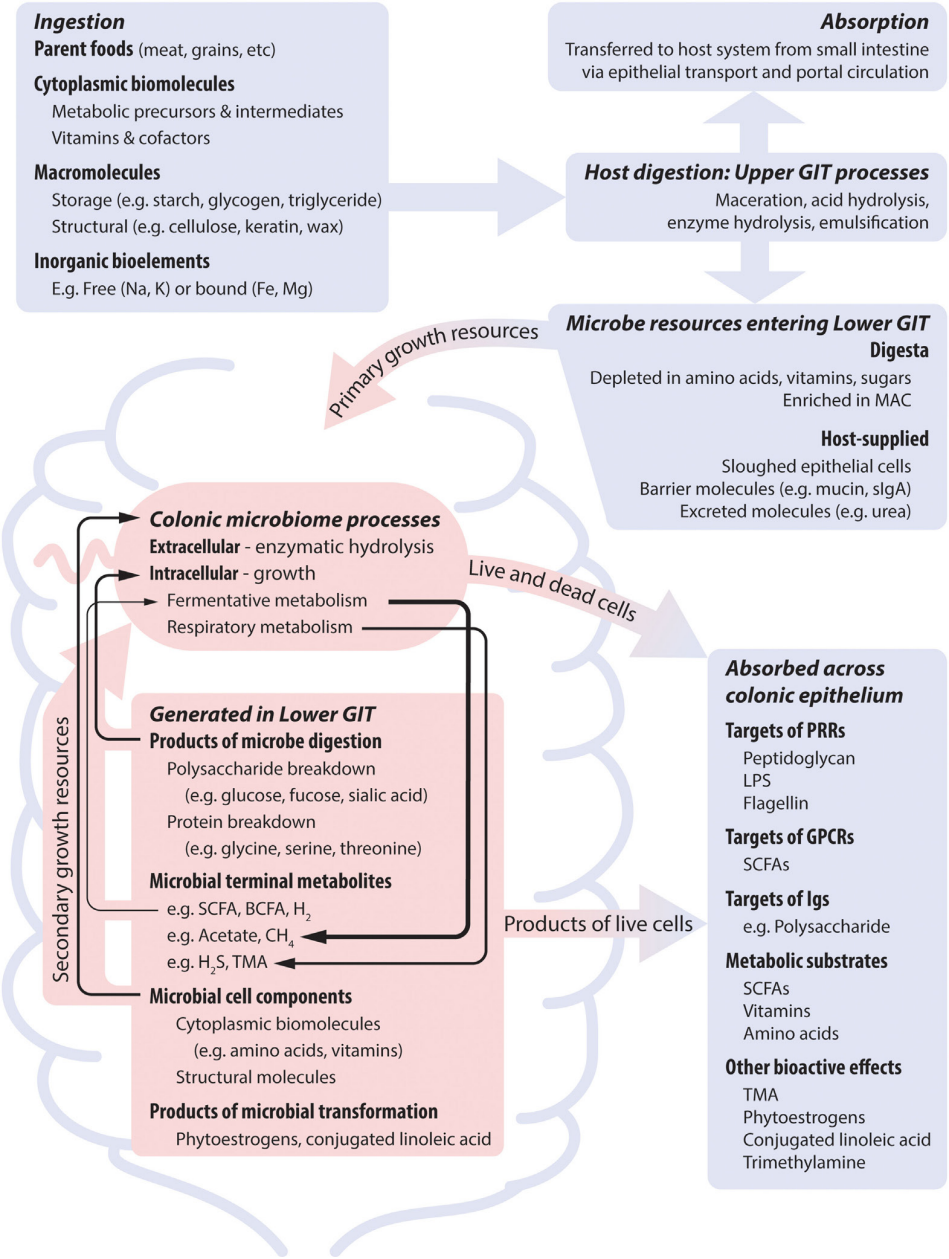
**Nutrient dimensions and host processes together shape microbial nutrient profile, and thus microbial growth and metabolic dynamics, which subsequently impact on the host system**. Blue and red areas represent host and microbial processes, respectively. The activity of the microbiome is supported by dietary nutrients that bypass host absorption and by host secretions. Temporal patterns result from differences in meal intervals and gastrointestinal motility. Many microbes are dependent on co-operative metabolic interactions to completely meet their nutrient requirements (indicated by black arrows). Metabolites that are the product of growth-related metabolism of dietary nutrients can be produced at high levels and may show positive feedback to diet. Metabolites that are produced by non-growth transformations of dietary components are produced at low levels if relevant microbes are present. Abbreviations: GIT, gastrointestinal tract; sIgA, soluble immunoglobulin A; MAC, microbe accessible carbohydrate; LPS, lipopolysaccharide; SCFA, short-chain fatty acid; BCFA, branch chain fatty acid; TMA, trimethylamine.

To extract nutrients from a macromolecule, all organisms must possess: (1) mechanisms to solubilize (digest) the source compound; (2) uptake systems to internalize the released/solubilized molecules; and (3) the biochemical capacity to metabolize the molecule. Humans show little variation in these capabilities, but microbial species differ considerably in all three. Consequently, the physical structure and chemical composition of food, together with host nutrient absorption, determine the dietary portion reaching ileal and colonic microbes (Figure 1). For instance, studies of ileostomy patients found that only 17% of dietary protein, 2% of digestible dietary starch, and <5% of dietary fat reaches the colon (25). Food processing/preparation also modulates host access to dietary nutrients. A total of 1% of milled-, yet 16% of flaked-dietary barley was recovered from ileostomy patients (25), and cooking reduces the α-amylase digestion-resistant portion of banana starch from 54 to 0% (26). Finally, small intestinal starch absorption capacity increases with slower transit times (27).

As such, the digesta entering the large intestines lacks easily absorbable organic compounds (e.g., sugars, central metabolites). Rather, it is enriched in digestion-resistant carbohydrate (fiber), predominantly non-starch polysaccharides, the microbiome’s dominant carbon, and energy source. The importance of microbe accessible carbohydrates (MACs) as niche dimensions driving microbial life history is seen by the diversity of carbohydrate-active enzymes that microbes possess (28–31). Nevertheless, MAC profile is not the only diet factor that strongly drives microbial community state. The colonic digesta is also depleted of electron acceptors (such as oxygen) to support respiratory metabolism, and pre-formed metabolic intermediates, especially nitrogenous compounds (e.g., amino acids, purine, pyrimidines). The low redox potential environment means that utilization of MAC is via the relatively energetically inefficient fermentative metabolism. Together with organic nitrogen compound depletion, this drives diverse strategies for meeting nitrogen requirements. Organisms can compete for limited organic nitrogen sources, adopt costly biosynthesis from inorganic nitrogen, or engage in co-operative cross-feeding interactions. Thus, MAC is typically highly available from a large number of sources, whereas nitrogen has low availability via few sources. As such, we postulate that dietary nitrogen content will have more predictable impacts on community that are global across all diet conditions, and that, conversely, alterations to MAC profile will impact a more select subset of microbes within a community, namely, those with the capacity to utilize the altered substrates.

Host physiology also influences the availability of microbial carbon/energy and nitrogen sources, largely through dead epithelial cells, mucin and uric acid/urea secretions. Intestinal epithelial cells secrete mucin glycoproteins to form a surface layer. Mucins have a bottlebrush-like structure comprising a serine and threonine rich protein backbone heavily decorated by glycan side chains (32). They constitute a carbohydrate and protein source to intestinal microbes, structurally distinct from dietary sources. A diverse range of microbes degrade mucins to access their sugars (e.g., fucose, sialic acid) and amino acids (1, 33–35). Uric acid and urea, host metabolic waste products, are also significant bacterial nitrogen sources. Although the host can impact microbial nutrition via these routes the outcome is interdependent with diet: host-excreted intestinal nitrogen increases with dietary protein content (36), and mucin-derived nutrients increase in importance under conditions of fasting or low caloric intake (23).

In summary, multiple diet dimensions influence processes impacting community state. Differences in food composition, meal sizes, and fasting periods create temporal variation in resource availability. Hence, the outcomes of microbial interactions, either competing or co-operating for access to resources, is constantly changing. Modeling studies support metabolic cooperation as a strong driver of co-occurrence patterns in diverse microbial communities (37) and demonstrate that heterogeneity in interaction strengths influences the emergence of stable states (7, 18). Chronic dietary changes extending over long time frames can induce the emergence of a distinct stable community underpinned by complex interaction networks. Hence, it is important to adopt an integrative perspective of diet. This suggests that cross-sectional studies of diet–microbiome interactions that test one of two nutrient dimensions will have limited explanatory power. Indeed such studies often have conflicting results (14). By adopting a nutritional geometry experimental design where macronutrient dimensions of diet are systematically varied (22, 24), we recently demonstrated that broad microbial responses to diet can be predicted at the macronutrient level (23).

## Microbiome Dimensions that Impact the Host

Microbially produced molecules (MPMs) exert a range of effects on the host: some represent nutritional resources, bioactive molecules influence developmental or regulatory networks, and effectors directly manipulate host cells. These categorizations are not mutually exclusive and an MPM can exhibit multiple effects.

Microbes contribute nutritional resources to the host primarily though short-chain fatty acids (SCFAs), vitamins, and amino acids. They contribute 10–15% of human energy requirements through SCFAs resulting from MAC fermentation (38, 39). Specific SCFAs have differing effects on the host; the major SCFAs acetate and propionate are transported to the liver and greatly impact energy balance, and as the primary colonocyte energy source butyrate supports epithelial function (40–42). Microbes differ in the SCFAs they can produce, and few species produce substantial quantities of propionate and butyrate (42, 43). In principle, microbial amino acid and vitamin generation can also supplement our nutrition where the diet is inadequate. While this has been established in many animals and is important for many herbivores, the conditions under which microbes support human nutrition in this way is unclear.

The host monitors SCFA levels through dedicated receptors that trigger a range of signaling pathways. Many of these pathways regulate the gastrointestinal environment, the microbial community (through immune function), or host metabolism. For example, SCFA-signaling on enteroendocrine cells mediates peptide YY secretion, a gut motility-inhibiting hormone (44). SCFAs also promote gut epithelial mucin secretion (45, 46). Finally, SCFA production is associated with inflammatory regulation through several routes, such as the promotion of Treg cell differentiation in the colon (47–49). These responses are sensitive to total and relative concentrations of SCFA types, and they collectively impact microbial activity and their interaction with the host by modulating the gut environment.

The host is sensitive to microbial structural components through a number of pathways. MAMPs such as lipopolysaccharide (LPS) and flagellin are targets for the TLR4 and TLR5 innate immune signaling pathways (50, 51). Although widely present in bacteria, structural variants of LPS and flagellin can trigger very different responses. Through lipid A core structural variation, LPS-expressing strains differ in their inflammatory capacity, which has been implicated in autoimmune disease triggering (52). Bacterial flagellin structural variation is thought to alter its interaction with TLR5 (51).

While the examples above concern MPMs common among gut microbes, some structural components are scarcely distributed among bacteria and can trigger distinct responses. For example, segmented filamentous bacteria closely associate with the mouse intestinal epithelium, and profoundly impact Th17 induction (53, 54). The polysaccharide A of *Bacteroides fragilis* has been shown to stimulate differentiation of Treg cells (55).

The above examples all involve fairly direct links between intrinsic properties of the microbes and homeostatic regulation of the host. As such, they may be viewed as points of co-ordination of host–microbiome interaction. However, there are also less controlled interactions. Metabolites produced through non-fermentative pathways by low-abundance microbes can influence host physiology and regulatory network signaling. These include deleterious metabolites such as trimethylamine (TMA) produced from choline (found in e.g., red meat, poultry, and eggs) degradation or H_2_S production from sulfate reduction (14, 56, 57). Microbial transformation of dietary components can also generate beneficial metabolites. For instance, microbes transform plant polyphenols into phytoestrogens thought to protect against breast and prostate cancer, cardiovascular disease, osteoporosis, and menopausal symptoms (58). Microbial conjugation of ω-6 fatty acids into conjugated linoleic acids increases insulin sensitivity, reduces adiposity and atherosclerosis, carcinogenesis, and has anti-inflammatory properties (59, 60).

In summary, predicting health impacts requires consideration of diet-microbiome dynamics. Diets supporting microbial growth that is beneficial to the host, e.g., through generation of SCFA, will likely cause positive host–microbiome feedbacks. Non-growth-related metabolites that depend on microbial activity (e.g., TMA, isothiocyanates) will be produced only if host diet contains the transformation substrates, and the transforming microbes are supported. Finally, for structural components such as MAMPs, the relative balance of microbes possessing distinct types will dictate inflammatory consequences. Nutritional geometry provides a powerful platform for exploring how dietary components interact to effect specific outcomes, but mechanistic-level modeling approaches are needed to explore how dietary inputs intersect with community interaction networks to support microbial activities ranging from community- to strain-specific levels. These models must include host–microbiome feedbacks also, as they are a critical factor in dictating which communities can develop and their resilience.

## Framework to Describe Diet–Microbiome Dynamics

As discussed above, microbes influence the immune system and host health *en masse* through mechanisms operating at different ecological scales. Some effects arise from clonal populations of pathogens (e.g., toxin production), representing microbial activity at a fine ecological scale. In contrast, SCFA production is a more complex and emergent outcome of many populations co-operating as a network. Thus, to describe the diet–microbiome–host axis, we must consider the microbiome’s properties at scales from individual cells to group activities of interaction networks. High-throughput technologies characterize specific community properties at the DNA, RNA, protein, and metabolite levels. Describing diet–microbiome–host dynamics requires integrating these datasets and linking predictions of cellular properties (their activity and impact on the host) with those of community state dynamics.

Critical cell properties to be extracted from high-throughput datasets include each microbe’s resource requirements (e.g., carbon and nitrogen sources), potential for MPM generation, and response to changing environmental dimensions. Microbe resource requirements and metabolic potential can be determined through pure cultures, or predicted from genome sequences. A community’s possession of these properties can then be inferred from high-throughput 16S amplicon or shotgun sequencing. These approaches generate lists of taxa or microbial properties present in a single timepoint sample. However to infer diet effects on host–microbiome interaction we must also consider response dynamics, which requires a more precise conceptual framework for community ecology.

**Figure 2:**
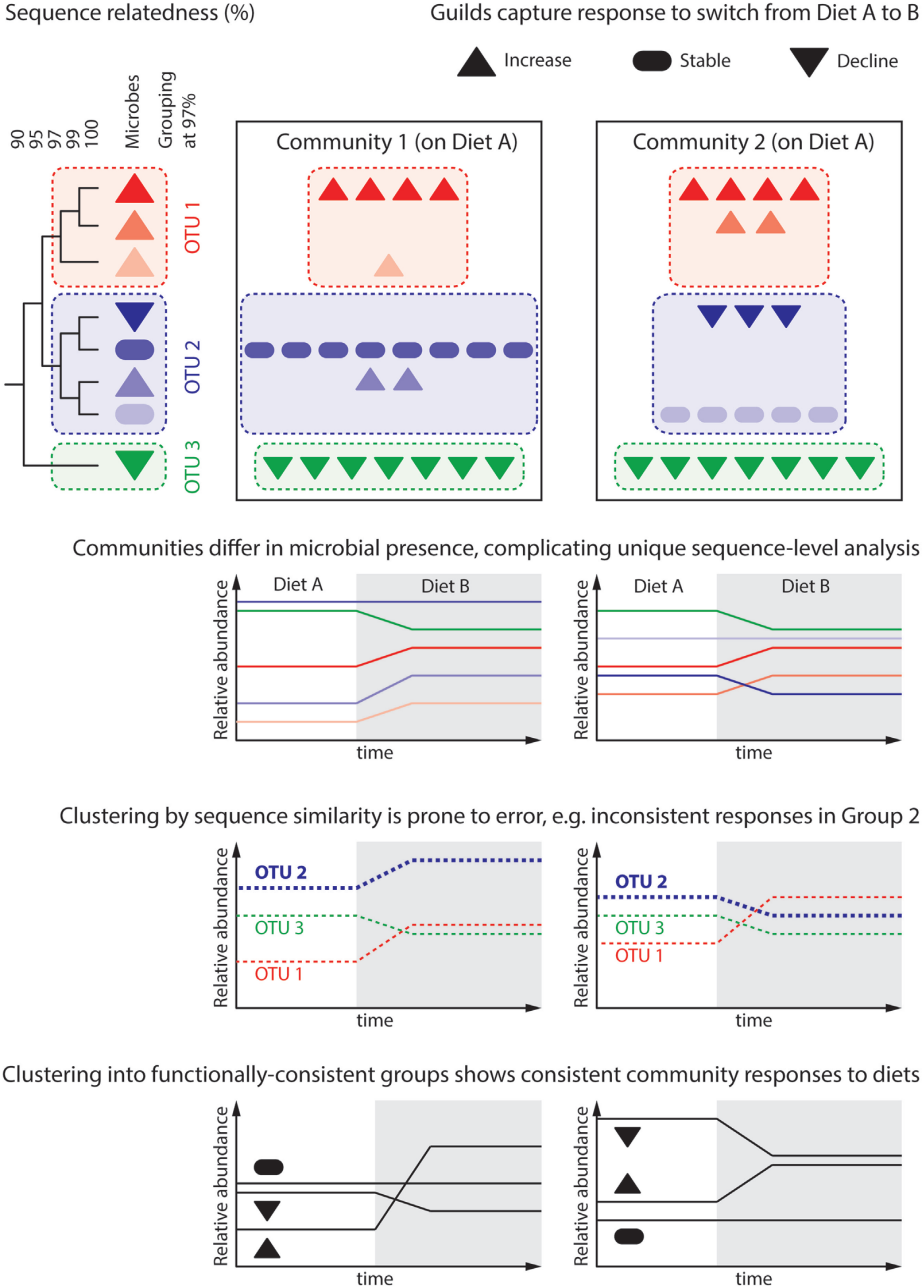
**Predicting the microbiome response to dietary intervention requires that we define “units of response” capturing groups of microbes similarly responding to environmental parameters**. The dendrogram depicts the genomic similarity of eight hypothetical microbes, which are differentially present in two communities. These communities are subject to a change in host diet, under which each microbe population either increases, declines, or remains stable. Attempts to reason about the community at the level of unique sequences are cumbersome as each community contains microbes that the other does not. This represents an over-classification, wherein several “units” (unique sequences) capture each possible response. Common practice in gut microbial ecology is to cluster unique sequences sharing 97% of their genomes into distinct groups, often termed “operational taxonomic units” (OTU). While analysis at the level of groups common to all communities can be performed, differential group constituent presence can still generate misleading results; this is because microbial traits, such as response to a given dietary change, do not correlate well with genomic similarity. For instance, Group 2 is seen to increase in Community 1, but decrease in Community 2. More meaningful is to group microbes by their response to environmental variables, in this case response to changing dietary intake: “*guilds*.” An open challenge for the microbial ecology field is predicting a microbe’s response to environmental variables based on either its genome or pure culture experiments.

Important concepts are populations, functionally similar population sets, and communities (Figure 2). Terms such as “strain/clone/ecotype/species” define *populations* of organisms deemed biologically equivalent and allow their distinction. Precisely defining populations occupying the same ecological niche maximizes a model’s predictive power. Populations do not differ equally, nor in the same way. This impacts their roles in a community, and conceptual grouping of functionally similar *population sets* aims to capture this. Examples include taxonomic groupings around similar characteristics, e.g., “genus” or “family,” and groupings of shared ecological or physiological properties, “guilds” or “functional types” (61). Accounting for functional complementarity or redundancy through population sets can improve ecological models. Finally, as reviewed above, the microbiome’s greatest health impacts arise as emergent properties. The *community type* concept aims to capture stable emergent property changes arising from self-stable networks of dissimilar populations. The gut microbiome’s adoption of distinct community types, termed *enterotypes*, was first proposed in Ref. (62). Although this has proven controversial, and the extent of distinction between proposed enterotypes is disputed, a rapidly emerging body of evidence demonstrates that microbial communities exhibit the property of multi-stability (7, 18, 63).

A recent study modulating the gut microbiome in obesity through diet revealed populations and guilds through co-abundance and co-occurrence patterns in metagenome data (64). Biologically equivalent bacteria populations with similar environmental responses should exhibit highly autocorrelated gene abundances. Thus, sets of sequence reads belonging to the same co-abundance group (CAG) represent a population’s pangenome (its constituents’ collective genomes). A similar analysis over CAGs (populations) can classify populations into sets termed genome interaction groups (GIGs) (64). Significantly, this approach revealed the lack of biological precision in several gut species. For example, nine different CAGs were taxonomically affiliated to *Faecalibacterium prausnitzii*. However, their distribution over several distinct GIGs suggested they have distinct ecological roles. Across this study, GIG abundances either remained stable, all declined or all increased in response to the diet modulation, consistent with the ecological guild concept. In summary, the ability to explain emergent outcomes of diet modulation was enhanced by describing the community in terms of interaction groups as well as species.

## Predictive, Integrative Modeling

The full potential of conceptually modeling the diet–host–gut microbiome axis lies in its integration with platforms that predict system responses to given interventions (65, 66). Statistical models constructed through machine learning have demonstrated that high-throughput microbiome analysis datasets contain the information and patterns required to predict an individual’s response to defined diets (20, 67). Though lacking mechanistic explanation, these models could reveal associations between the conceptual units explored above if those abstractions can be applied to high-throughput datasets prior to their input. Making abstractions that generalize across datasets and experiments is a key challenge and will become a focus of the field in the coming years.

Fine-scale metabolic networks are already being reconstructed through mechanistic genome scale models (68–70). Built upon organisms’ annotated genomes, these models are predicting community growth and metabolic responses to given nutritional environments. We anticipate that in the coming years these models will be coupled with agent-based and mathematical modeling frameworks to facilitate the integration of host–feedback pathways. The more abstractive agent-based (23, 71), partial- and ordinary-differential equation models (7, 18, 72) are ideal for capturing the conceptual units explored above, and their mechanistic relationships. Deployment within a nutritional geometric framework that systematically varies dietary inputs to these models can reveal how specific dimensions of diet together interact with intrinsic features of microbes and their potential for interactions with one another and the host to drive community states.

Predictive, integrative models applied to complex systems can help tame complexity and identify core principles governing system function. In the coming years, we anticipate the development of integrative models highlighting how concerted alterations to dietary composition, intake patterns, and administration of pre-, pro-, and antibiotics can together shift a stable microbial community to another desired state. This is the key to providing personalized, tailored manipulations for specific patients that shift their microbiomes from disease- to health-promoting states.

## Ethics Statement

MR is supported by the David and Judith Coffey Life Lab philanthropic donation, made to the Charles Perkins Centre. The donors were not involved in any aspect of this study.

## Author Contributions

Both MR and AH conceived and wrote the manuscript.

## Conflict of Interest Statement

The authors declare that the research was conducted in the absence of any commercial or financial relationships that could be construed as a potential conflict of interest.
